# Bioactive compounds produced by gut microbial tannase: implications for colorectal cancer development

**DOI:** 10.3389/fmicb.2014.00684

**Published:** 2014-12-05

**Authors:** Félix López de Felipe, Blanca de las Rivas, Rosario Muñoz

**Affiliations:** Laboratory of Bacterial Biotechnology, Institute of Food Science, Technology and Nutrition – Spanish National Research Council (ICTAN-CSIC), MadridSpain

**Keywords:** microbial tannase, bioactive compounds, colorectal cancer, gallic acid, pyrogallol

## Abstract

The microorganisms in the human gastrointestinal tract have a profound influence on the transformation of food into metabolites which can impact human health. Gallic acid (GA) and pyrogallol (PG) are bioactive compounds displaying diverse biological properties, including carcinogenic inhibiting activities. However, its concentration in fruits and vegetables is generally low. These metabolites can be also generated as final products of tannin metabolism by microbes endowed with tannase, which opens up the possibility of their anti-cancer potential being increased. Patients with colorectal cancer (CRC) display an imbalanced gut microbiota respect to healthy population. The recent use of next generation sequencing technologies has greatly improved knowledge of the identity of bacterial species that colonize non-tumorous and tumorous tissues of CRC patients. This information provides a unique opportunity to shed light on the role played by gut microorganisms in the different stages of this disease. We here review the recently published gut microbiome associated to CRC patients and highlight tannase as an underlying gene function of bacterial species that selectively colonize tumorous tissues, but not adjacent non-malignant tissues. Given the anti-carcinogenic roles of GA and PG produced by gut tannin-degrading bacteria, we provide an overview of the possible consequences of this intriguing coincidence for CRC development.

## INTRODUCTION

An imbalance in intestinal bacteria potentially leading to diseases termed colonic dysbiosis is associated to CRC, however, the possible role of intestinal microorganisms in the different CRC-stages is currently under debate.

Host diet is a crucial variable to shape the gut microbiota and a risk factor for CRC. Increased fruit and vegetable consumption is a dietary habit modification aimed to prevent gastrointestinal cancer (WHO Technical Report Series, No. 916 [TRS 916], 2003). Plant foods are main sources of tannins, dietary phytonutrients that display pro-apoptotic and antimetastasic properties in animal and *in vitro* studies (Serrano et al., 2009). Despite its promising chemopreventive potential, results from human studies to evaluate the association between the intake of foods rich in tannins and cancer risk are more dissuading. For example, inverse association (Theodoratou et al., 2007) but also no association (Kyle et al., 2010) between dietary proanthocyanidin intake and CRC risk have been reported. These apparently conflicting results may reflect interindividual differences in gut microbiota and reveal the difficulty to infer biological effects from dietary intake records without considering tannin-microbiota interactions. The microorganisms in the human gastrointestinal tract have a profound influence on the transformation of food into metabolites which can impact human health (Nicholson et al., 2012). Thus, the highly individual gut microbial activity, in particular the tannin-metabolizing activity of CRC-associated microbiota, will probably determine the bioavailability and physiological effects of tannins and their degradation products on tumorous tissues.

## TANNASE: A COMMON FUNCTION IN THE MICROBIOTA COLONIZING COLORECTAL TUMOROUS TISSUES

Gut microbial tannase activity is likely crucial to determine the tannin-metabolizing phenotype of CRC-associated microbiota. Tannase (tannin acylhydrolase) transforms the gallate esters of tannins and other phenolic compounds, such as epigallocatechin gallate, into GA. GA can, subsequently, be decarboxylated by gallate decarboxylase to yield PG as final product of tannin metabolism. The capacity for tannin degradation is a survival advantage over other gut microorganisms because tannins display antimicrobial activity (Chung et al., 1998), reduce the species richness in the gut (Tan et al., 2013) and probably improve the adaptation of tannin-degrading bacteria to the intestinal environment (Reverón et al., 2013).

Recently the CRC microbiome encompassing all DNA within the microbiota of late stage CRC patients has been disclosed (Tjalsma et al., 2012). This information enabled these authors to flesh out the microbiota that colonizes tumorous or adjacent non-malignant sites. If one focuses on these intestinal microorganisms and perform a search for homologous genes to the known *Lactobacillus plantarum* tannase gene (KEGG database http://www.genome.jp/kegg/), only bacterial spp. specifically overrepresented in tumorous tissues, but not in adjacent non-malignant tissues, display tannase-homologous genes (**Table 1**). This intriguing coincidence could be consistent with the selectivity of plant phenolic compounds to target cancer cells (Azmi et al., 2013) and suggest that the presence of gut microbial tannase activity may have potential implications on the colonic epithelial health.

**Table 1:** Gut microbial tannase in late stage CRC-patients.

On tumor^a^	Organism	Tannase	Gene^b^
*Roseburia*	*R. intestinalis* XB6B4	+	rix:RO1_26850
Streptococcaceae	*Streptococcus gallolyticus* ATCC 3143	+	sgt:SGGB_1624
	*S. gallolyticus* ATCC BAA-2069	+	sgg:SGGBAA2069_c16370
	*S. gallolyticus* UCN34	+	sga:GALLO_1609
	*S. gallolyticus* UCN34	+	sga:GALLO_0933
	*S. gallolyticus* ATCC 3143	+	sgt:SGGB_0917
	*S. gallolyticus* ATCC BAA-2069	+	sgg:SGGBAA2069_c09070
	*S. gallolyticus* ATCC BAA-2069	+	sgg:SGGBAA2069_c09080
*Fusobacterium*	*F. nucleatum* subsp. *vincentii*	+	fnc:HMPREF0946_00654
	*F. nucleatum* subsp. *nucleatum* ATCC 25586	+	fnu:FN0616
	*F. nucleatum* subsp. *animalis*	+	fus:HMPREF0409_00517
*Aggregatibacter*	*A. actinomycetemcomitans* D7S-1	+	aan:D7S_02361
	*A. actinomycetemcomitans* D11S-1	+	aat:D11S_1302
	*A. aphrophilus*	+	aap:NT05HA_0741
	*A. actinomycetemcomitans* ANH9381	+	aao:ANH9381_1645
Coriobacteriaceae	*Slackia heliotrinireducens*	+	shi:Shel_22280
	*Collinsella*	NA		
	*Coriobacterium glomerans*	-		
Peptostreptococcaceae		NA		
**Off tumor^a^**								
Enterobacteriaceae	*Citrobacter*	-		
	*Shigella*	-		
	*Cronobacter*	-		
	*Kluyveria*	NA		
	*Serratia*	-		
	*Salmonella*	-		
*Ruminococcus*		-		
*Clostridium*^c^		+		
*Anaerovorax*		NA		
*Eubacterium*		-		
**On/Off tumor^**a**^**								
*Bacteroides*		-		
*Faecalibacterium*		-		

*^a^On tumor: bacteria inhabiting tumorous tissues; Off tumor: bacteria inhabiting adjacent healthy tissues; On/Off tumor: bacteria inhabiting indistinctly tumorous and adjacent healthy tissues.*

*^b^Homologous genes to *L. plantarum* tannase lp_2956.*

*^c^Two *Clostridium* spp. (*C. botulinum* E3 and *C.* strain SY8519) displayed genes with homology to *L. plantarum* tannase. However, this spp. are not reportedly associated to colorectal malignancies.*

*NA. Sequence not available.*

## IMPORTANCE OF TANNASE-GENERATED METABOLITES IN CRC DEVELOPMENT

Recent findings have highlighted the potent DNA-damaging activities of tannins and tannase-derived metabolites, including PG, GA, and epigallocatechin gallate (Hossain et al., 2013). The genotoxic behavior of these metabolites is determined by its pro-oxidant activity which is in line with the pro-oxidant activity displayed by a number of further anti-cancer phenolic compounds in cellular/biological systems (Azmi et al., 2013).

Although the antioxidant capacity of tannins, PG and GA, is generally accepted, its pro-oxidant behavior should not be surprising. Thus, plants elaborate these natural phenolics to have, among other important functions, antimicrobial activity and antimicrobials share a common mechanism to kill bacteria: through the overproduction of ROS (Kohanski et al., 2007).

It is to be noted that the pro-oxidant behavior of tannase-derived metabolites in CRC-tissues can be boosted in the colon compared to the small intestine due to a more pronounced pro-oxidant environment in the former (Sanders et al., 2004). The presence of cooper, a transition metal participating in the Fenton-like chemistry, is another factor for inducing the chemopreventive, pro-oxidant behavior of certain plant phenolic compounds (Azmi et al., 2013), including GA (Hossain et al., 2013), especially when it has been found in particularly augmented concentrations in a number of malignancies (Linder, 2012). Nevertheless some phenolics, such as PG, do not necessarily require a metal ion catalyst to produce OH radicals (Hossain et al., 2013).

Given the pro-oxidant activity of plant phenolics has been proposed as one of its anti-cancer properties (Azmi et al., 2013), the question arises as to whether the production of GA and PG by tannase-producing CRC-associated bacteria affects CRC development. There are several hints that the answer is yes. The potent ROS generation by GA and particularly PG strongly activates the tumor suppressor p53 in RKO CRC cells (Hossain et al., 2013). In addition GA and PG are known glutathione depletors (Park et al., 2007; You and Park, 2010) and could directly exert a cancer cell killing action via potent ROS generation. Hence, the selective production of GA/PG by the CRC-associated microbiota could promote pro-oxidative stress in the tumorous tissues to increase the direct or apoptotic killing (via p53 activation) of cancerous cells. Consistent with this hypothesis recent findings have suggested that tannase-mediated biotransformation of green tea extracts or epigallocatechin gallate *in vitro* modulates the expression of several pro and anti-apoptotic genes to enhance the pro-apoptotic behavior of these natural compounds (Macedo et al., 2012). Although details on these regulatory effects were not reported, an increased bioavailability of GA produced by tannase could account for the observed differences. Besides influencing apoptotic processes, GA modulates the expression of genes involved in cell cycle, angiogenesis, and metastasis inducing death in various cancer cell lines (Verma et al., 2013). Among other processes GA significantly modulates NF-κB, Akt, and ATM kinase signaling pathways to prevent the processes of carcinogenesis and it is a competitive inhibitor of the pro-inflammatory mediator COX-2.

## ENHANCING THE ANTI-CARCINOGENIC POTENTIAL OF GALLIC ACID AND PYROGALLOL BY THE GUT TANNIN-DEGRADING BACTERIA COLONIZING COLORECTAL TUMOROUS TISSUES

Gallic acid and PG display anticancer properties, however, it must be noted that its concentration in fruits and vegetables is generally low (Tomas-Barberán and Clifford, 2000). Hence the tannic acid transformation performance by gut tannin-degrading bacteria will likely play a key role to increase the concentration and bioactivity of GA and PG in the colon. The bioaccumulation of GA and PG in microbial biofilms may be another factor contributing to increase the anti-carcinogenic effects of these bioactive compounds. In this respect, biofilm formation on the collagen-rich surfaces accessible in the displaced mucosal epithelium of carcinomas is the most outstanding characteristic of *Streptococcus gallolyticus*, a tannin-degrading bacterium unquestionably associated to CRC (Tjalsma et al., 2012). This bacterium has been proposed to increase, via adhesion, colonization of tumorous tissues by members of *Fusobacterium* spp. [another bacterium with capacity to produce tannase (**Table 1**)] which in turn own capacity to adhere to other bacterial ssp. and attract them to the tumor (Tjalsma et al., 2012). According to this colonization pattern and given bacterial taxa of the microbiome colonizing colon tumor tissues generally own the capacity to produce tannase (**Table 1**), it is conceivable to speculate on the formation of multispecies biofilms on cancerous tissues settled by bacteria able to process dietary tannins. Microbe-generated GA and PG accumulated in these biofilms may thus selectively increase its bioavailability and bioactivity in the tumorous tissues. While this scenario is probably correct for tumorous tissues of late stage CRC patients, it is doubtful to anticipate the same in early and adenomatous stages as the occurrence of tannase-producing opportunistic pathogens in the healthy human gut, such as *S. gallolyticus* or *Fusobacterium* spp., is very rare (Tjalsma et al., 2012). In this regard, unraveling the microbiomes associated to the early and adenomatous stages of CRC will be crucial to define the specific role of tannase-producing bacteria in CRC development. In addition, the identification of the gut microbiota in CRC patients in epidemiological studies addressing the association between dietary tannin intake records and tumor recurrence or regression, may be critical in understanding the role of gut bacteria on the anti-cancer effects of dietary polyphenols.

## CONCLUSION

Tannase is an underlying gene function of gut microorganisms that selectively colonize tumorous tissues of late stage CRC patients. Based on the anti-carcinogenic inducing roles reported for GA and PG, the observed colonization pattern suggests that these gut tannin-degrading bacteria are involved in the prevention rather than promotion of tumor progression. Tannase then could be submitted as a link in the yet elusive relationship between dietary factors, CRC-associated gut microbiota and CRC progression. A drawback to this hypothesis is that the ROS-mediated anti-cancer effects of the GA and PG produced by the tannase-producing bacteria naturally colonizing tumorous tissues could be blocked by the high levels of ROS-destroying antioxidants usually possessed by the cancerous cells. Nevertheless, therapeutic microbiology strategies considering the affinity of tannase-producing bacteria for colonic tumorous tissues could be a powerful weapon to efficiently produce ROS-generating drugs that selectively kill CRC cells.

## Conflict of Interest Statement

The authors declare that the research was conducted in the absence of any commercial or financial relationships that could be construed as a potential conflict of interest.

## ABBREVIATIONS

CRC: colorectal cancer
GA: gallic acid
PG: pyrogallol
ROS: reactive oxygen species
